# Influence of Initial Moisture Content on Heat and Moisture Transfer in Firefighters' Protective Clothing

**DOI:** 10.1155/2017/9365814

**Published:** 2017-03-30

**Authors:** Dongmei Huang, Song He

**Affiliations:** ^1^College of Quality and Safety Engineering, China Jiliang University, Hangzhou, Zhejiang 310018, China; ^2^Key Laboratory of Furniture Inspection Technology of Zhejiang Province, Hangzhou, Zhejiang 310018, China; ^3^State Key Laboratory of Fire Science, University of Science and Technology of China, Hefei, Anhui 230027, China

## Abstract

This paper presents a model for heat and moisture transfer through firefighters' protective clothing (FPC) during radiation exposure. The model, which accounts for air gaps in the FPC as well as heat transfer through human skin, investigates the effect of different initial moisture contents on the thermal insulation performance of FPC. Temperature, water vapor density, and the volume fraction of liquid water profiles were monitored during the simulation, and the heat quantity absorbed by water evaporation was calculated. Then the maximum durations of heat before the wearer acquires first- and second-degree burns were calculated based on the bioheat transfer equation and the Henriques equation. The results show that both the moisture weight in each layer and the total moisture weight increase linearly within a given environmental humidity level. The initial moisture content in FPC samples significantly influenced the maximum water vapor density. The first- and second-degree burn injury time increase 16 sec and 18 sec when the RH increases from 0% to 90%. The total quantity of heat accounted for by water evaporation was about 10% when the relative humidity (RH) is 80%. Finally, a linear relationship was identified between initial moisture content and the human skin burn injury time before suffering first- and second-degree burn injuries.

## 1. Introduction

Firefighters' protective clothing (FPC) constitutes critically important equipment in firefighting. Typical FPC consists of two parts: an outer shell and an inner linear [1, 2]. In general, the moisture content in FPC material significantly affects the wearer's thermal and moisture comfort. These effects have sparked considerable interest in researching the mechanisms of heat and moisture transfer in FPC materials [3–5].

Moisture contained in FPC material usually results from human sweat, penetration of outside water during a rescue, or moisture in the settled environment [6–8]. With sufficient heat, liquid moisture in the fabric undergoes an endothermic process and transforms into steam [9]. In addition, moisture in the fabric increases the heat capacity of the material, such that moist materials absorb more heat. Therefore, moisture in the fabric can limit the amount of energy transferred to human skin. Recent research has examined the effects of moisture that comes from human sweat and outside water [10–12].

The initial moisture content of FPC material is determined by the relative humidity (RH) of the storage environment, and moisture generally settles in the pores of the fabric before heating. Moisture evaporation mainly occurs during the early stage of heating, which is of critical importance in determining heat and moisture transfer. In this paper, we describe a simulation approach that can characterize the influence of initial moisture content on the heat insulation performance of multilayer FPC materials with air gaps between each layer. The intention of this study is to describe moisture absorption, desorption, and evaporation during the early stage of heating. In order to explain the mechanism by which initial moisture content acts to influence the thermal protective performance of these materials, we also estimate the maximum duration of the temperature before the wearer suffers first- and second-degree burn injuries.

## 2. Experimental Methods

**Figure 1:**
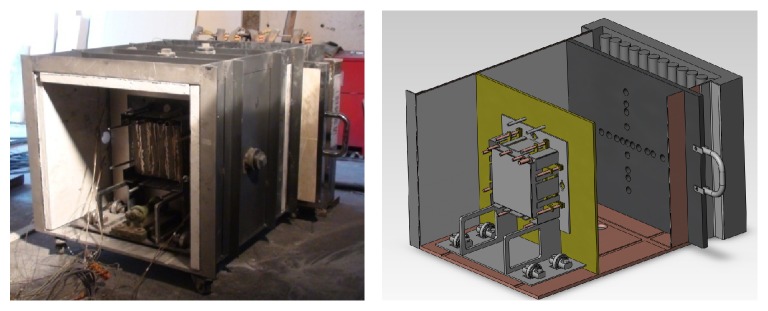
Schematic diagram and view of experimental chamber.

The experimental equipment used in this study consisted of a radiation source, test chamber, constant temperature system, and data acquisition system, as shown in Figure 1. A detailed introduction to the experimental equipment can be found in He et al.'s paper [13].

The samples were cut from the FPC currently in use in China. The FPC material is composed of four layers: the outer shell (OS), the moisture barrier layer (MB), the thermal barrier layer (TB), and the comfort layer (CL). The materials comprising the layers are XDB602, aramid fabric coated with PTFE film, aramid insulation, and cotton shirting, respectively. The dimension of the samples was 230 mm × 230 mm. First, eight samples were set in a drying oven (ZK-1000A, Shanghai Hong Yun experimental equipment factory, China) at a temperature of 100°C for 24 h to remove internal moisture. Then two of the samples were put into programmable constant temperature and humidity testers (PCTHC) for 24 h. The temperature of the PCTHC was set at 20°C, and the humidity was set at 30 ± 5%, 50 ± 5%, 65 ± 5%, and 80 ± 5% to achieve different initial moisture contents among the samples. The humidity allowed moisture to be wicked into layered samples and held in the fiber pores [14], leading to an increase in moisture content. The moisture weight in each fiber layer was determined after the sample was placed into different relative humidity situations using an electronic balance with an accuracy of 0.001 g over three measurements.

**Figure 2:**
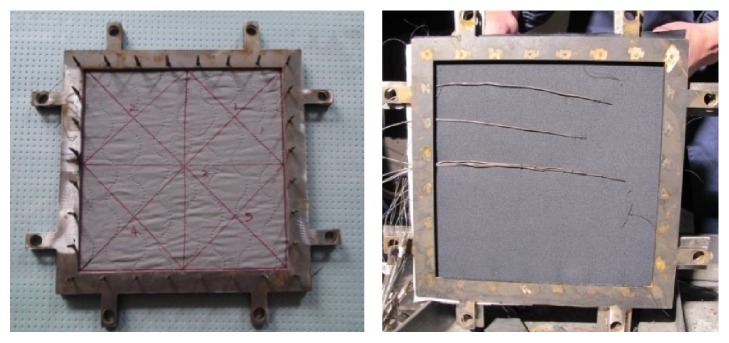
Thermocouples and sample holder.

The thermocouples used were identical to those described in He et al.'s paper [13], and we named them thermocouple at the outer shell layer (TCO), thermocouple at the moisture barrier layer (TCM), thermocouple at the thermal liner layer (TCH), thermocouple at the comfort layer (TCC), and thermocouple at the wearer skin surface (TCW), as shown in Figure 2. The samples were fixed in the test chamber using a sample holder on the sample trolley. According to Lawson's report [15], the radiation intensity on the corridor floor decreases as the distance from the fire source increases, and the radiation intensity is about 1.8−10 kW m^−2^. In this experiment, the radiation intensity was set at a constant value of 5 kW m^−2^. We measured the temperature at the outer surface of the outer shell layer, which increased gradually. The radiation source was turned off after 120 sec because the FPC material began combustion and totally lost its thermal insulation performance after 120 sec under 10 kW m^−2^.

## 3. Theoretical Models

**Figure 3:**
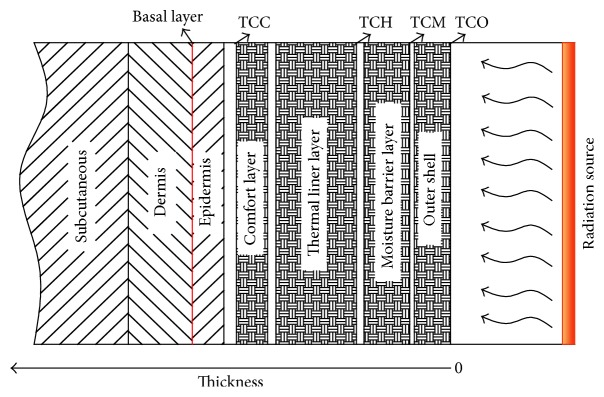
Sketch of heat and moisture transfer in FPC materials and heat transport in human skin.

This study developed a model for coupled heat and moisture transmission in FPC materials during the radiation heating process. The effects of both initial moisture content as determined by RH and the temperature of the storage environment on the FPC samples' insulation performance were investigated. The burn injury times for human skin under different conditions were also determined.  Figure 3 shows a sketch of heat and moisture transfer in FPC materials, as well as heat transfer through human skin. The structure and materials of each layer were simulated in the experiment. In practice, air gaps (AG) exist between each fabric layer of FPC material, because the four fabric layers are separated by a small amount of space. Therefore, our computer simulation incorporated an AG of 0.5 mm between each fabric layer. There is also an air gap between the comfort layer and the wearer's skin, which has a thickness of 1 mm. The human skin consists of three layers, from outermost to innermost: epidermis, dermis, and subcutaneous.

We based our simulation model on Chitrphiromsri's model [10, 16, 17], which can simulate heat and moisture transfer in multilayer fabric. This simulation is built upon the equations for mass, momentum, and energy conservation. The conservation equations in each fabric layer are as follows.

Energy equation is as follows:
  (1) ρcpeff∂T∂t+ρcplvl∂T∂x=∂∂xkeff∂T∂x+Δhvapm˙vl+m˙vb+Δhtransm˙lb+m˙vb+γq˙rade−γx+Q˙ii.
Mass equations are as follows:
  (2) solid:  ∂∂tρbεb=m˙vb+m˙lb,liquid:  ∂∂tρlεl+∂∂xρlvl=m˙vl−m˙lb,gas:  ∂∂tρvεg=∂∂xDeff∂ρv∂x−m˙vl−m˙vb.
Momentum equation for liquid water is as follows:
  (3) $$\begin{array}{c} v_{l}=-\frac{s\cdot K_{l}}{\mu_{l}}\frac{\partial }{\partial x}\left(\;P_{c}\;\right). \end{array}$$

In (1), ${\dot{Q}}_{ii}$ is the radiation heat flux between each fabric layer, defined as follows:

(4) Q˙ii=γqii−˙e−γx−∑0ii−1Lii+γqii+˙e−γ∑0iiLii−xii=1,2,3,4,

where *ii* is the layer number. For example, *ii* = 1 represents the outer shell layer, so $\dot{q_{ii}^{-}}$ and $\dot{q_{ii}^{+}}$ are the heat flux on the back and front surfaces, respectively. In this case

(5) qii−˙=δε~Tii−14−Tii4ii=1,2,3,4qii+˙=δε~Tii4−Tii+14ii=1,2,3,4.

The conservation equations for each air gap are as follows:

Energy equation is as follows:
  (6) $$\begin{array}{c} {\left(\;\rho c_{p}\;\right)}_{eff}\frac{\partial T}{\partial t}=\frac{\partial }{\partial x}\left(\;k_{eff}\frac{\partial T}{\partial x}\;\right)+\Delta h_{vap}{\dot{m}}_{vl}. \end{array}$$
Mass equation is as follows:
  (7) $$\begin{array}{c} \frac{\partial }{\partial t}\left(\;\rho_{v}\;\right)=\frac{\partial }{\partial x}\left(\;D_{eff}\frac{\partial \rho_{v}}{\partial x}\;\right)-{\dot{m}}_{vl}. \end{array}$$

The heat transfer in each skin layer was estimated using Pennes' model as follows [18]:

(8) ρCPskin∂T∂t=∇kskin∇T+ρCpbloodwbTart−T.

Initial conditions are as follows:
  (9) Tx,0=293Kρvx,0=ρv0,εlx,0=εl0,εbx,0=εb0,vlx,0=vl0.
Boundary conditions for the fabric are as follows:
  (10) Tx=0=−345.713e−t/39.1541+658.2739,−Deff∂ρv∂xx=0=hm,ambρv,amb−ρvx=0,vlx=0=0,εlx=0=εlleft,εbx=0=0,Tx=L=40C°,ρvx=L=0,vlx=L=0,εlx=L=0,εbx=L=0.

The temperature of the boundary conditions for the fabric is the fitting curve of the average temperature, as measured by the thermocouples under radiation of 5 kw m^−2^.

We used Henriques' tissue burn injury model [19] to determine the thermal damage to human skin under the radiation heating process. The thermal damage rate was estimated using the first-order Arrhenius rate equation. Human skin sustains a burn injury when the temperature of the basal layer (see Figure 3) rises above 44°C. In this case

(11) dΩdt=0,T<44C°Pexp⁡−ΔERT,T≥44C°.

Equation (11) can be expressed as an integral equation; namely,

(12) $$\begin{array}{c} \Omega =\overset{t}{\underset{o}{\int }}P\exp\left(\;-\frac{\Delta E}{RT}\;\right)dt. \end{array}$$

In this work, differential equations (1)-(2) and (6)–(8) were solved using the finite volume method, and time-stepping was carried out according to the well-known Crank-Nicholson method [20]. This process yielded a system of nonlinear algebraic equations, which was resolved by the goal chasing method. The conductivity and diffusivity on the interface between the air gap and the fabric layer were estimated using the harmonic mean. The under relaxation procedure with a parameter of 0.6 was utilized to prevent divergence of the iteration method. The iterations were repeated until the changes in the solutions became smaller than 10^−6^.

**Figure 4:**
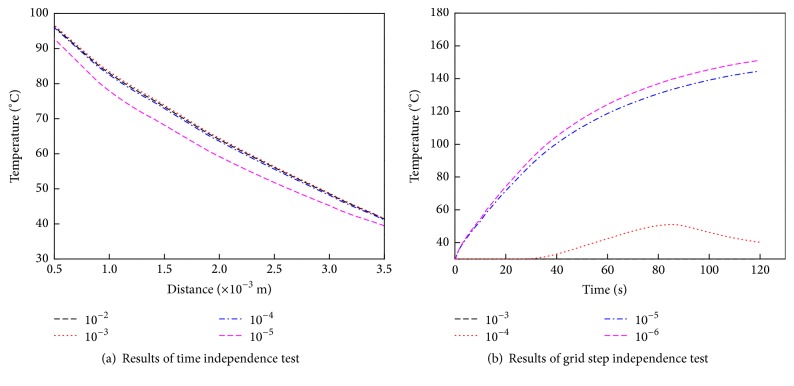
Temperature distributions for time and grid independence tests.

Time and step independence are illustrated graphically in Figure 4.  Figure 4(a) shows the temperature distribution in the fabric layers at 10 sec, and Figure 4(b) shows the temperature distribution at the mid thickness of the fabric layers. The data show that after a 10^−3^ time step, the results do not change significantly. CPU time increases from about 600 sec to 3,600 sec in an Intel i7-2600 processor when the time step increases from 10^−3^ to 10^−5^. The temperature distribution when the grid size exceeds 10^−4^ m is much different from that when the grid size is smaller than 10^−5^ m. The maximum temperature difference using a grid size of 10^−5^ m and 10^−6^ m is about 6°C. CPU time increases from about 3,600 sec to 21,600 sec in an Intel i7-2600 processor when the grid step decreases from 10^−3^ to 10^−6^. To summarize, the time and space step are 10^−3^ sec and 10^−5^ m, respectively.

## 4. Results and Discussion

### 4.1. Experimental Results

**Figure 5:**
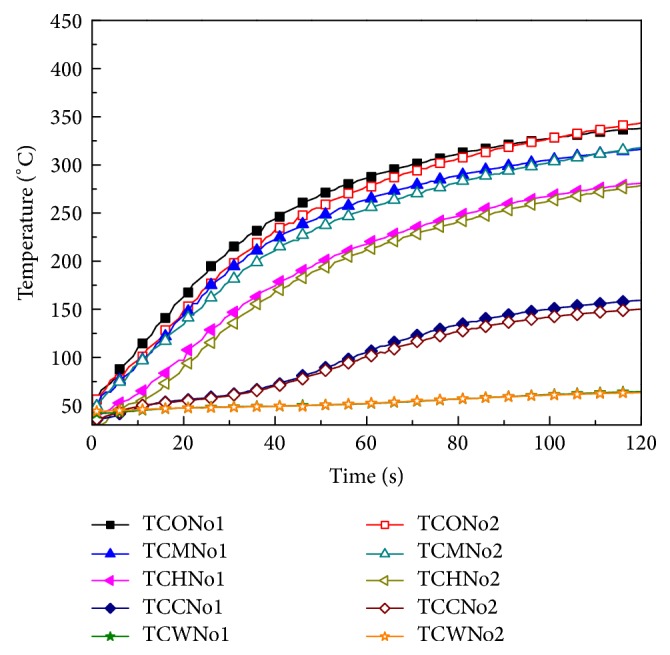
Temperature change with time at different locations.

To ensure the accuracy of the experimental results, we first analyzed experimental repeatability. The radiation intensity of the experiment was 5 kW m^−2^. The sample was dry; the AG thickness between the back surface of the fabric layer and human skin was zero. Other conditions were set as described above. The results are shown in Figure 5. The data demonstrate that the repeatability of experiment results is good.

**Figure 6:**
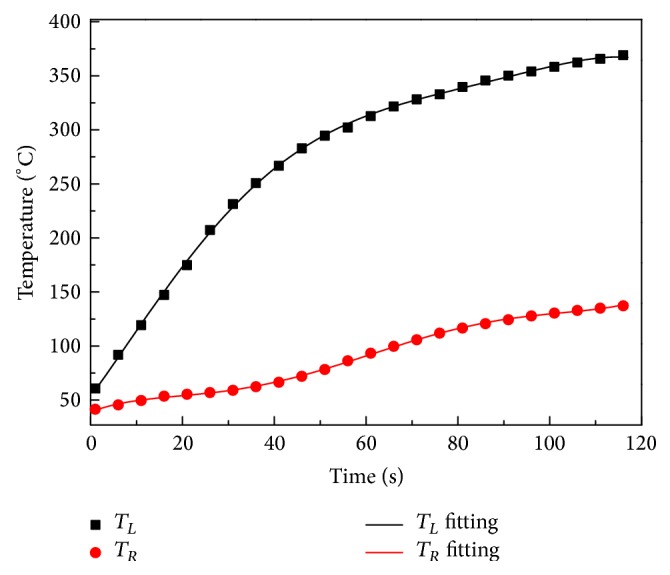
Temperature profiles at outer surface and back surface of fabric layer. The fitting equations for *T*_*L*_ and *T*_*R*_ are *T*_*L*_ = −345.713exp⁡(−*t*/39.1541) + 658.2739 and *T*_*R*_ = 311.96812 + 1.64134*t* − 0.07761*t*^2^ − 0.07761*t*^3^ + 0.00203*t*^4^ − 1.97183 × 10^−5^ + 6.46652 × 10^−8^*t*^5^.

In the simulation, the temperature measured at the outer surface and at the back surface of the fabric layer under a radiation intensity of 5 kW m^−2^ was set as the* T*0 and *T*_*L*_ boundary temperatures, respectively. The temperature equation was obtained by liner fitting using Origin software, as seen in Figure 6.

### 4.2. Simulation Results

**Figure 7:**
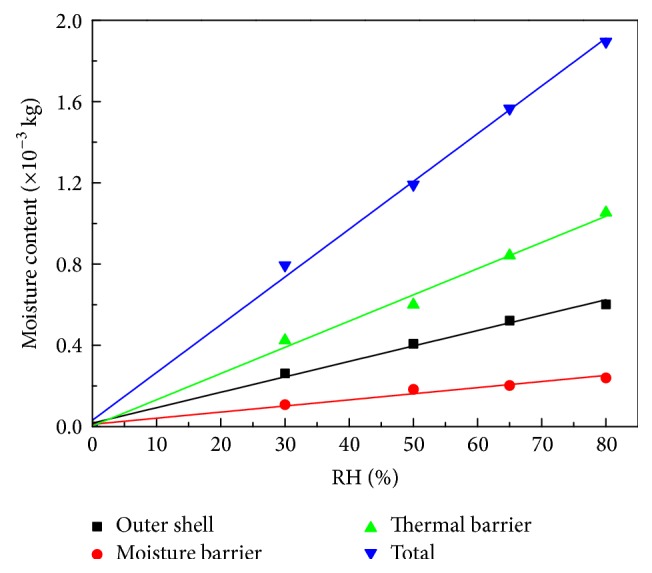
Moisture weight in each fiber layer against RH, with fitting line.

The thermal physical properties of the fabric samples used in the simulations can be reviewed in Chitrphiromsri et al. [17, 21]. The parameters for specific heat capacity, viscosity, density, and thermal conductivity of air at different temperatures are in Tao's paper [22].  Table 1 shows the thermophysical and geometrical properties of the fabric, and Table 2 provides the properties of human skin. The parameters of the Henriques equations were set identically to those in Liu et al.'s paper [23]. The thickness of each sample was determined by measuring with a caliper three times. The initial moisture content is shown in Figure 7.

The correlation coefficients of the fitting lines for the experimental data describing the outer shell, moisture barrier, thermal linear, and total multilayer material are 0.99205, 0.97086, 0.99226, and 0.99700, respectively. These results demonstrate that the moisture weight in each layer, as well as the total moisture weight, increases linearly with settled environmental RH, as expected. The moisture absorbed by wicking into the thermal barrier is about four times that in the outer shell layer and two times that in the moisture barrier layer. The samples also felt obviously moist when touched after remaining for 24 h in circumstances with RH values exceeding 65%.

**Figure 8:**
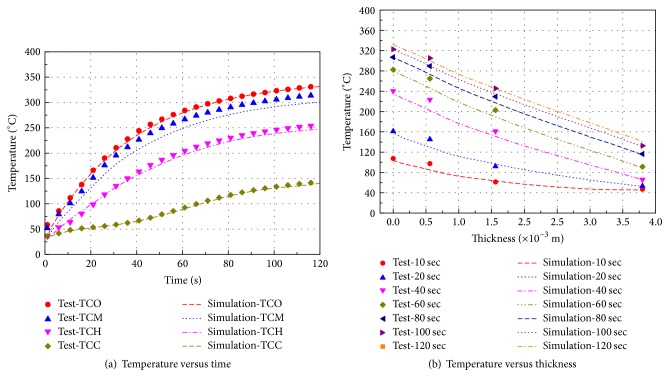
Simulated (continuous lines) and experimental (discrete points) temperature profiles when RH = 65%.

We designed an apparatus to simulate the process of heat transfer in FPC samples with different initial moisture contents. The experimental setup and the samples were described in Section 2. To ensure simulation accuracy, we compared the simulation results with the experimental measurements, which were corrected before analysis for radiation loss. The comparison model did not consider human skin. The boundary conditions were set identically to the experimental measurements.  Figure 8 shows the simulated (continuous lines) and experimental (discrete points) temperature profiles at RH = 65%. In the figure, TCO, TCM, TCH, and TCC represent the measurement results at different locations (as seen in Figure 3), while labels preceded by “*S*” represent the simulation results. The data show that the simulation results are in good agreement with the experimental results. The mechanism for heat and moisture transfer in a system consisting of FPC materials, air layers, and human skin with different initial moisture content conditions were then analyzed using the validated model combined with Pennes' model. The first and second human injury burn times were estimated using the Henriques' tissue burn injury model.

**Figure 9:**
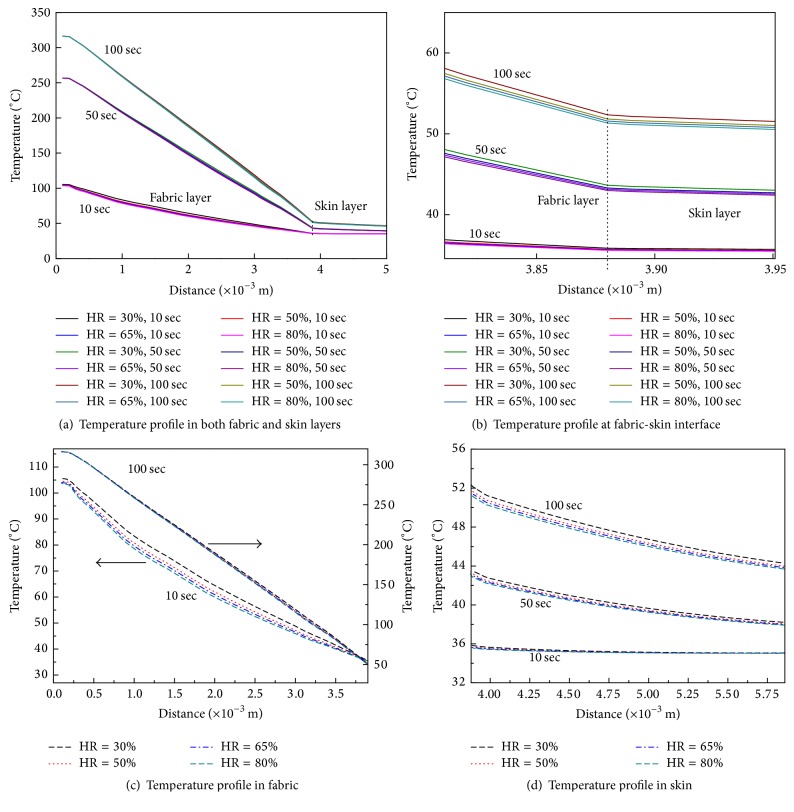
Temperature profiles in different layers at specific moments in time.

Figure 9 shows the temperature profiles in different layers at particular moments in time. The origin of the *x*-axis is at the outer shell surface, as shown in Figure 3.  Figure 9(a) shows that the temperatures in the fabric layers decrease significantly with distance at different moments in time, but temperatures decrease slowly in the skin layer. A clean break is identifiable between the fabric layer and the skin layer. This may be because heat transfer in fabric layers occurs through heat conduction, radiation, and water evaporation and heat absorption, while the only heat transfer model in the skin layer is heat conduction. The temperature difference in skin under different initial moisture contents is larger at 100 sec than at 10 sec, as shown in Figure 9(b). This is because less initial moisture content in the samples leads to more heat accumulated in the fabric and the air gap during radiation.

We then analyzed the temperature differences under different initial moisture conditions in the fabric and skin layers, with the results shown in Figures 9(c) and 9(d). The temperature difference in the fabric under different initial moisture contents is higher at 10 s than at 100 s. These results are in agreement with the accepted point of view that the initial moisture content of a sample affects heat transfer in the fabric primarily at the beginning of heating. The human skin begins at 0.00388 m. The skin surface temperature increases from 309°C at 10 sec to around 324°C at 100 sec under different initial moisture contents. The skin temperature increases as the initial moisture content of the fabric decreases.

**Figure 10:**
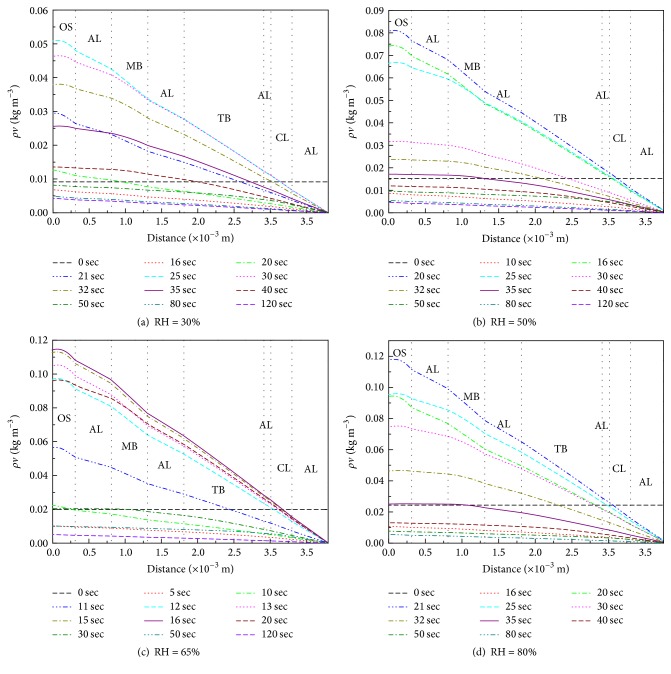
Distributions of water vapor density at different times.

**Figure 11:**
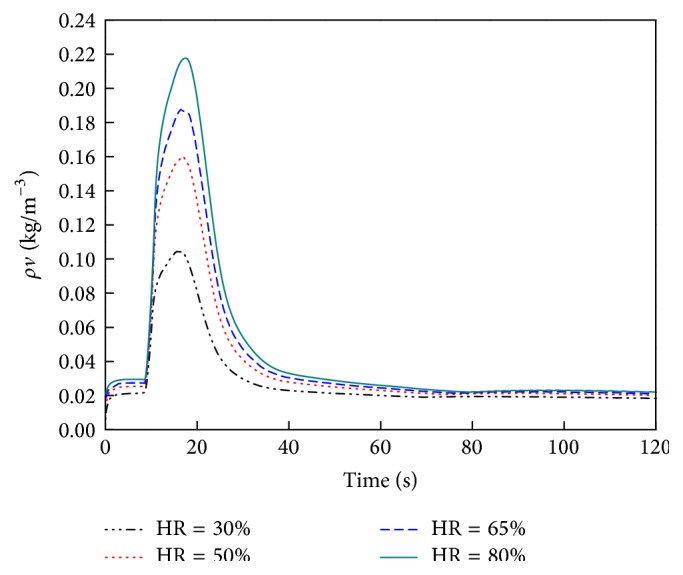
Profiles of water vapor density versus time at a distance of 0.56 × 10^-3 ^m.

Figure 10 shows the profiles for water vapor density in the fabric and air gap layers at different times. The data indicate that the water vapor density decreases as distance from the outer surface increases; this occurs because the water vapor density decreases with temperature, which decreases with distance. Higher temperatures provide more energy for water evaporation. The maximum water vapor density in each layer increases very rapidly with* t*, reaching a maximum at about 20 sec under the conditions outlined in this paper, as shown in Figure 11. The curve follows a normal distribution between about 10 sec and 35 sec. After 35 sec, the water vapor density decreases more slowly with time. In other words, the actuation duration of the initial moisture of FPC samples was less than 35 sec in this experimental situation.

**Figure 12:**
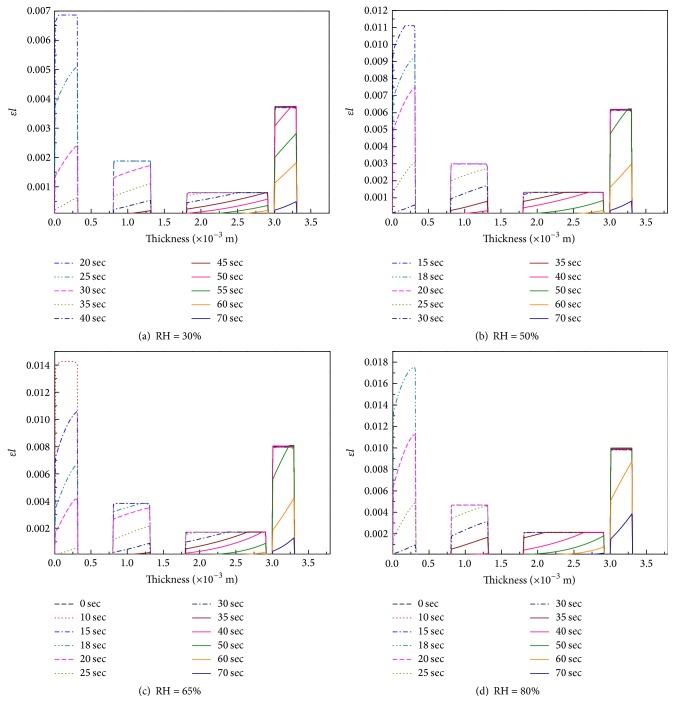
Liquid water volume fraction distributions at different times.

In our experiment, the temperature increased when the outer shell of the FPC sample was exposed to heat radiation, which caused the desorption and evaporation of bound water as well as the evaporation of free liquid water located in the fabric pores. To examine further the mechanism of moisture transformation during heating, we analyzed the liquid water volume fraction in the fabric pores at different times, as shown in Figure 12. The data show that the distribution of liquid water in the fabric, unlike that of water vapor, is completely noncontinuous. The initial liquid water volume fraction in each fabric layer is uniform, and it increases noticeably with increasing initial moisture content. As the temperature increases, the liquid water volume fraction decreases and the water vapor density increases.

**Figure 13:**
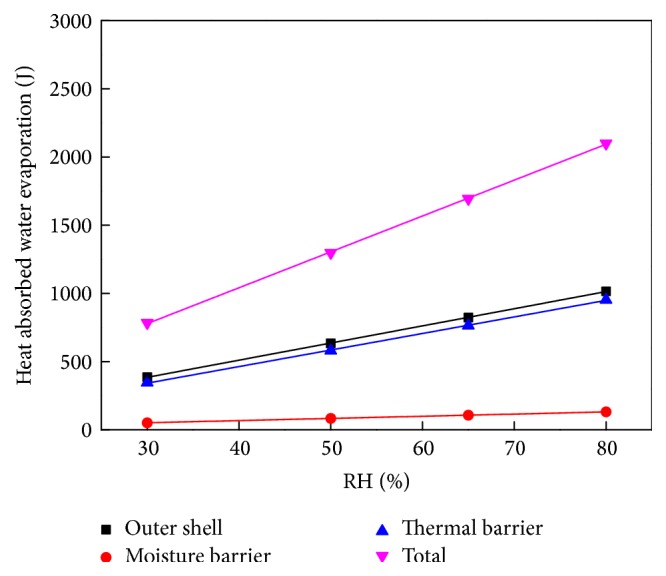
Heat absorbed by water evaporation against RH, with fitting line.

Figure 13 plots the heat absorbed by water evaporation against RH, with a fitting line. According to the conservation equations, moisture in the fabric consists of bound, liquid, and gaseous water. Under high temperatures, the bound water transfers to a liquid state, and the liquid water evaporates to a gaseous state. The enthalpy of the transition from bound water to free liquid water is about 250.9 J g^−1^ under relative humidity of 65%. The enthalpy of evaporation per unit mass is about 2400 J g^−1^. The bound water and liquid water volume fractions in each fabric layer can be derived from the simulation. Then the heat absorbed through water evaporation can be determined based on different initial moisture conditions, as shown in Figure 13. The data demonstrate that the heat absorbed by water evaporation increases linearly with increasing RH.

Figure 12 shows that the time required for the moisture to evaporate completely is about 70 s. The heat flux on the material surface is 5 kW m^−2^, and the dimension of the sample is 230 cm × 230 cm. The total quantity of heat applied to the surface of the material is about 18,515 J. Figure 13 shows that, at the RH level of 80%, the heat absorbed through water evaporation is about 2,000 J, which constitutes more than 10% of the total heat.

**Figure 14:**
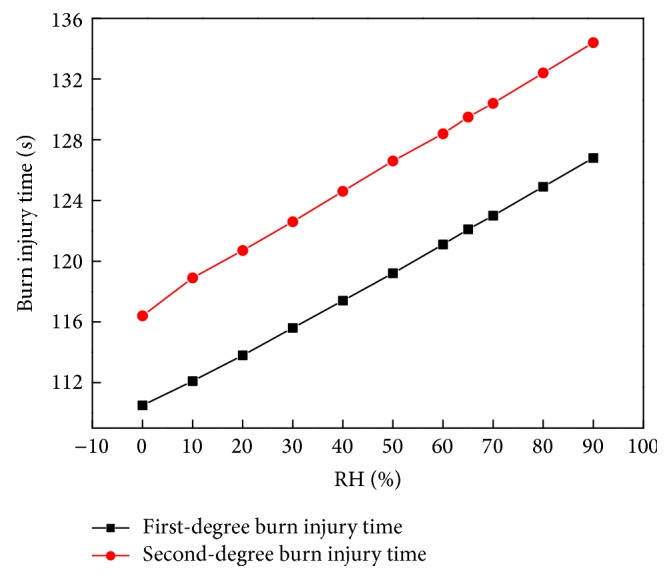
Burning injury time versus RH.

**Figure 15:**
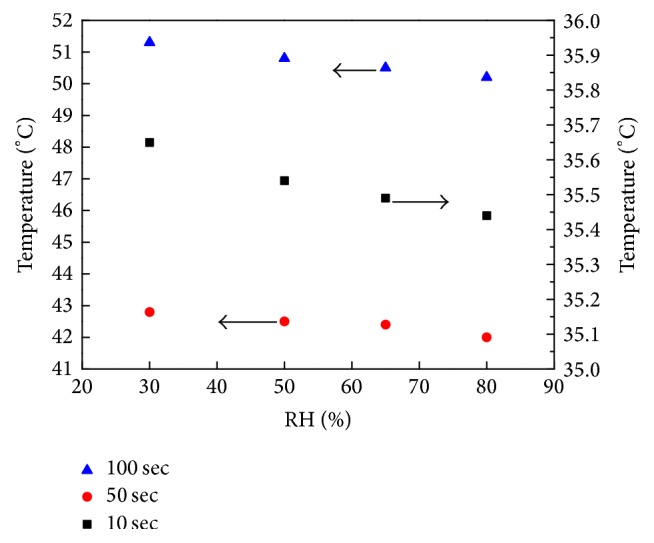
Temperature at skin surface.

We also investigated the effect of the initial fabric moisture content on the maximum duration of heat radiation before the wearer sustains first- and second-degree burn injuries. The tissue burn injury model is based on work by Henriques [19], and it is calculated according to (11). Takata's [24] criterion was used to determine that first- and second-degree burn injuries occur when *Ω* = 0.53 and 1, respectively, on the basal layer of the skin (the interface of the epidermis and dermis).  Figure 14 shows human skin burn injury time versus RH. The data show that, as the initial moisture content in the fabric increases, the maximum duration of heat radiation before acquiring first- and second-degree burn injuries increases linearly. This occurs because higher initial moisture in fabric pores allows more energy to be absorbed by water phase transfers during the heating process. The first- and second-degree burn injury time increases 16 sec and 18 sec when the RH increases from 0% to 90%. The heat absorbed through water evaporation is 10% of the total quantity of heat applied to the surface of the material. However, the comfort of clothing is usually very low when the clothing is set at the environment in which the RH is more than 80%. Therefore, the RH is usually less than 80%. The temperature at the skin surface at different moment is shown in Figure 15. We can see that the temperature decreases with increasing the initial moisture content. The maximum temperature difference between the RH of 30% and 80% condition is about 1 K, which is really slight. But the effect of energy on the skin injury is an accumulation behavior, which leads to the second-degree burn injury time increasing about 8 sec.

## 5. Conclusion

We developed a numerical model to investigate the effects of initial moisture content at each fabric layer on heat and moisture transfer in FPC materials. We estimated burn injury times under different initial moisture contents using our model combined with Pennes' model and Henriques' tissue burn injury model. We can draw the following conclusions:

1. Initial moisture content in each FPC fabric layer increases linearly as the RH of the storage location increases.
2. We developed a comprehensive model to simulate heat and moisture transfer in FPC, and the simulation results show fairly good agreement with the experimental results.
3. Water vapor is distributed both in the fabric layers and in the air layer, while free liquid water disperses only in the fabric layers. Water vapor density is mainly due to evaporation from free liquid water during the experiment, and it decreases as initial moisture content increases. The total amount of heat absorption attributable to water evaporation is about 10% when the RH is 80%.
4. A linear relationship exists between the initial moisture content and the maximum duration of heat radiation before causing first- and second-degree burn injuries.

## Figures and Tables

**Table 1:** Thermophysical and geometrical properties of fabric [17, 21, 22].

Property	Outer shell	Moisture barrier	Thermal barrier	Comfort layer
Thickness of the material *L*^*ii*^ [m]	0.31 × 10^−3^	0.5 × 10^−3^	1.1 × 10^−3^	0.29 × 10^−3^

Material of composition	XDB602	Aramid fabric coated with PTFE film	Aramid insulation	Cotton shirting

Density *ρ*_*f*_ [kg m^−3^]	677.42	210.00	77.27	413.79

Thermal conductivity (*k*)_*f*_ [W m^−1^ K^−1^]	0.075	0.052	0.05	0.053

Volume fraction *ε*_*f*_	0.334	0.186	0.115	0.15

Fibre curl *τ*	1.5	1.25	1	1

Diffusivity of the gas phase in the fabric *D*_*f*_ [m^2^ sec^−1^]	0 × 10^−14^	0 × 10^−14^	0 × 10^−14^	0 × 10^−14^

Fiber radius *d*_*f*_ [m]	1.6 × 10^−5^	1.6 × 10^−5^	1.6 × 10^−5^	1.6 × 10^−5^

Darcian permeability coefficient, m^2^ *K*_*l*.sat_ [m^2^]	10 × 10^−16^	10 × 10^−16^	10 × 10^−16^	10 × 10^−16^

Saturation of the fabric *s*_*ir*_	0.1	0.1	0.1	0.1

Proportional constant of liquid water absorption *γ*_*ls*_ [kg m^−3^]	0 × 10^−4^	0 × 10^−4^	0 × 10^−4^	0 × 10^−4^

**Table 2:** Thermophysical and geometrical properties assigned to skin layers [14, 15, 25–28].

Property	Epidermis	Dermis	Subcutaneous tissue	Blood
Density *ρ*_skin_ [kg m^−3^]	1200	1200	1000	1060

Thermal conductivity (*k*)_skin_ [W m^−1^ °C^−1^]	0.23	0.45	0.19	—

*L*_skin_ [m]	0.08 × 10^−3^	2 × 10^−3^	10 × 10^−3^	—

*c*_*p*skin_ [J kg^−1^ °C^−1^]	3600	3300	2300	3770

*ω*_*b*_ [m^3^ sec^−1^ m^−3^ tissue]	0	0.00125	0.00125	—

## Symbols

*c*_*p*_: Specific heat at constant pressure, J kg^−1^ °C^−1^
*D*: Diffusivity of the gas phase in the fabric, m^2^ sec^−1^
*d*_*f*_: Fiber radius, m
*h*_*m*_: Convective heat transfer coefficient, W m^−2^ °C^−1^
Δ*h*: Enthalpy per unit mass, J kg^−1^
*ii*: Fabric layer
*k*: Thermal conductivity, W m^−1^ °C^−1^
*K*: Darcian permeability coefficient, m^2^
*L*: Thickness of the material, m
${\dot{m}}_{vl}$: Mass transfer rate from the gaseous phase to the liquid phase, kg m^−3^ sec^−1^
${\dot{m}}_{vb}$: Mass transfer rate from the gaseous phase to the solid phase, kg m^−3^ sec^−1^
${\dot{m}}_{lb}$: Mass transfer rate from the liquid phase to the solid phase, kg m^−3^ sec^−1^
*P*_*c*_: Pressure of liquid water, Pa
${\dot{q}}_{rad}$: Incident radiation heat flux from the radiation onto the outer fabric surface, W m^−2^
$\dot{q_{ii}^{-}}$: Radiation heat flux from the *ii* + 1 fabric layer to the *ii* fabric layer, W m^−2^
$\dot{q_{ii}^{+}}$: Radiation heat flux from the *ii* − 1 fabric layer to the *ii* fabric layer, W m^−2^
${\dot{Q}}_{ii}$: Total radiation heat flux of the *ii* fabric layer, W m^−2^
*s*: Saturation of the fabric
*t*: Time, s
*T*: Temperature, °C
*v*: Velocity of liquid water, m sec^−1^
*x*: Distance, m

### Greek Symbols

$\tilde{\tau }$: Transmissivity of the fabric
*τ*: Fiber curl
*ρ*: Density, kg m^−3^
$\tilde{\varepsilon }$: Absorption coefficient
*ε*_*b*_: Volume fraction
*γ*: Radiative extinction coefficient of the fabric, m^−1^
*μ*_*l*_: Dynamic viscosity, kg m^−1^ sec^−1^
*σ*: Stefan-Boltzmann constant, *δ* = 5.6705 × 10^−8^ W °C^−4^ m^−2^
*ω*_*b*_: Blood perfusion [0.00125 m^3^ sec^−1^ m^−3^ tissue]
*Ω*: Quantitative measure of the burn damage at the basal layer of human skin
*P*: Frequency factor or preexponential factor, sec^−1^
Δ*E*: Activation energy for skin
*R*: Universal gas constant, 8.315 × 10^3^ J kmol^−1^ °C^−1^

#### Subscripts

0: Initial state
eff: Effective
*l*: Liquid water
*b*: Bound water
*v*: Water vapor
skin: Skin
blood: Blood
amb: Ambient air
vap: Evaporation
trans: Transfer
art: Human core
*L*: Left boundary
*R*: Right boundary.
